# Supplementary material for: Cubam receptor-mediated endocytosis in hindgut-derived pseudoplacenta of a viviparous teleost (Xenotoca eiseni)
Source: J Exp Biol. 2021 Jul 2;224(13):jeb242613. doi: 10.1242/jeb.242613 (PMC8278012; doi:10.1242/jeb.242613)
Supplement: Supplementary information [file jexbio-224-242613-s1.pdf]

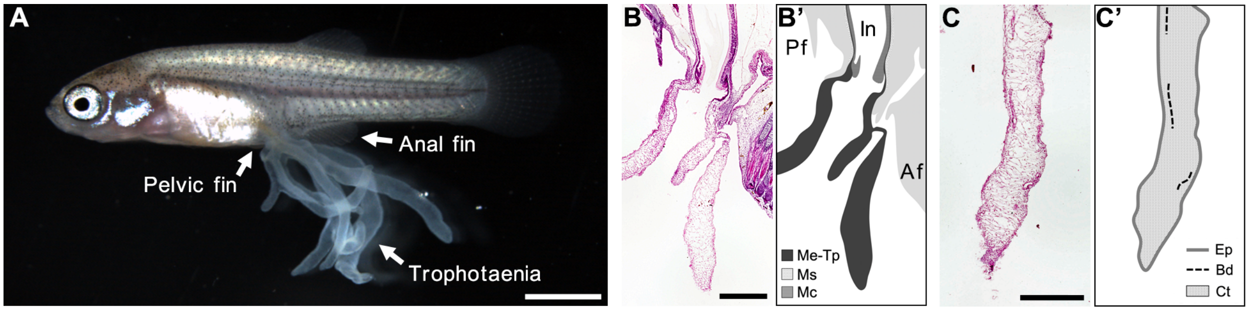

Figure S1. Tissue structures of the trophotaenia in *Xenotoca eiseni* embryos.

A. Whole body photograph of *X. eiseni* 4th week embryo. The trophotaeniae are elongated from surround of the anus located between the pelvic fin and anal fin. Scale bar, 2 mm. **B**. Hematoxylin and eosin (HE)-stained section (B) and corresponding illustration (B') of the tissue structure around the anus at the border of the hindgut and trophotaenia of the embryo at the fourth gestational week. Trophotaeniae are sequential to the mesenchymal layer of the intestine. In, intestine; Pf, pelvic fin; Af, anal fin; Me-Tp, mesenchyme-to-trophotaenia; Ms, muscle layer; Mc, mucosal layer. Scale bar: 500 μm. **C**. HE-stained section (C) and corresponding illustration (C') of the internal structure of the trophotaenia. Ep, epithelial layer cells; Bd, blood vessel; Ct, connective tissue. Scale bar: 500 μm.

Journal of Experimental Biology • Supplementary information

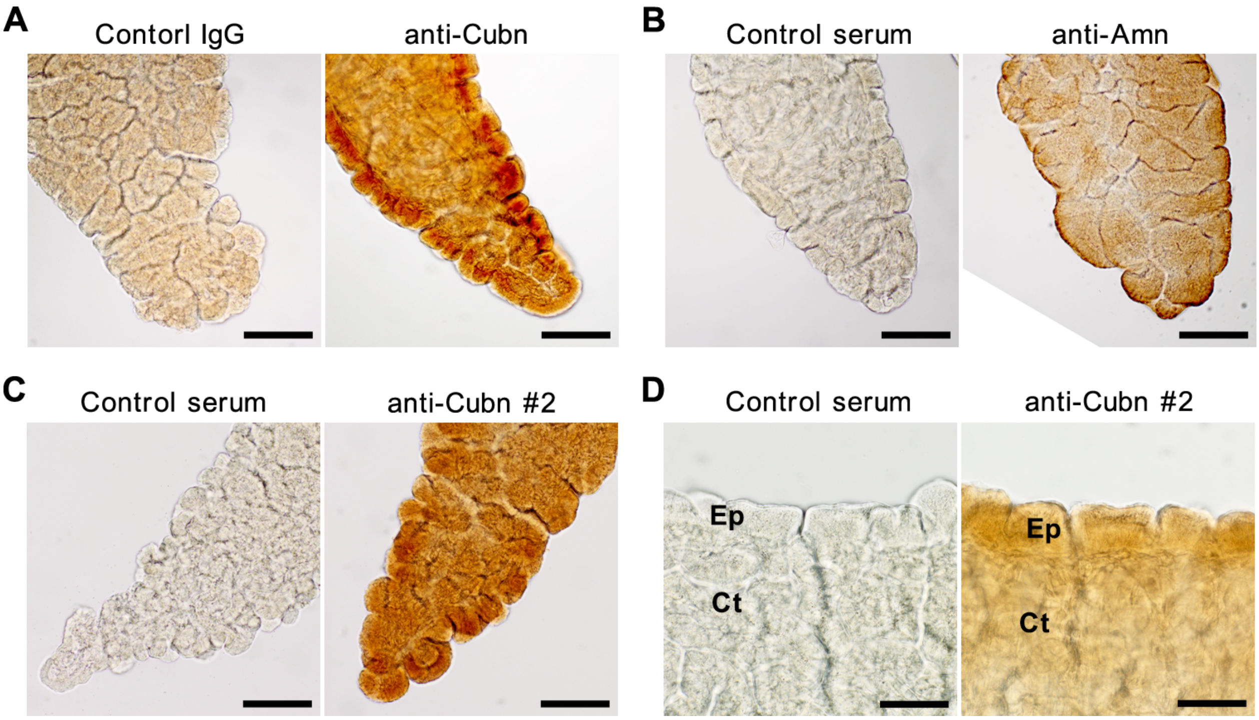

Figure S2. Immunohistochemistry of Cubn or Amn in the trophotaenia.

A-B. Immunohistochemistry using the Cubn antibody (A) or Amn antiserum (B) in the apical terminal of the trophotaenia. Scale bar: 100 µm. **C-D**. Immunohistochemistry using the Cubn antiserum in the apical terminal (C) or the epithelial layer (enlarged image) (D) of the trophotaenia. Ep, epithelium layer; Ct, connective tissue. Scale bar: 100 µm (C) or 50 µm (D).

Journal of Experimental Biology • Supplementary information

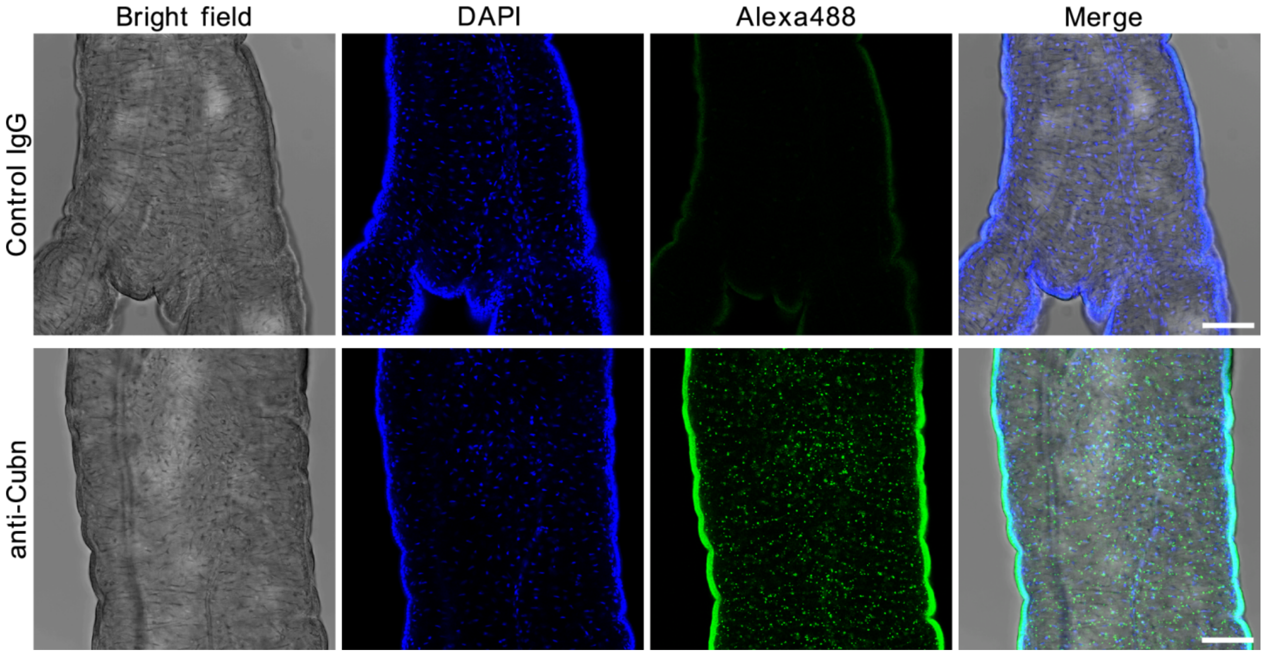

Figure S3. Fluorescent immunohistochemistry using Cubn antibody in the trophotaenia.

Confocal microscopy analysis of the trophotaenia of *Xenotoca eiseni* embryos at the fourth week of gestation. Anti-Cubn signals are observed in the epithelial layer cells of the trophotaenia. DAPI, 4′,6-diamidino-2-phenylindole. Scale bar: 20 μm.

Journal of Experimental Biology • Supplementary information

```
H.sapiens DAB2 (NP_001334.2) MSNEVETSATN-GQPDQ---------------QAAPKAPSKKEKKKGPEK 34
X.eiseni  dab2               MSTEVENSVPATADPSSPSTASTTISPTTSPVTTTPKAQLKKEKKKVPEK 50
                             **.***.*..  .:*..                ::***  ****** ***

H.sapiens DAB2 (NP_001334.2) TDEYLLARFKGDGVKYKAKLIGIDDVPDARGDKMSQDSMMKLKGMAAAGR 84
X.eiseni  dab2               TDEYLLSRFQGDGVRYKAKLIGIDDVSEARGDKMCQDSMMKLKGMAVAAR 100
                             ******:**:****:***********.:******.***********.*.*

H.sapiens DAB2 (NP_001334.2) SQGQHKQRIWVNISLSGIKIIDEKTGVIEHEHPVNKISFIARDVTDNRAF 134
X.eiseni  dab2               SQGKHKQRIWVNISMSGLKIIDEKSGVIEHEHVVNKISFIARDVTDNRAF 150
                             ***:**********:**:******:******* *****************

H.sapiens DAB2 (NP_001334.2) GYVCGGEGQHQFFAIKTGQQAEPLVVDLKDLFQVIYNVKKKEEEK-KKIE 183
X.eiseni  dab2               GYVCGAEGQHQFFAIKTAQQAEPLVIDLKDLFQVIFNMRKKEAESSQKAE 200
                             *****.***********.*******:*********:*::*** *. :* *

H.sapiens DAB2 (NP_001334.2) EASKAVENGSEALMILDDQTNKLKSGVDQMDLFGDMSTPPDLNSPTESKD 233
X.eiseni  dab2               NGSAVVENGG-ALQSTDGES-KAAQPVEQLDLFGDITTPPDIRAP----- 243
                             :.* .****. **   *.:: *  . *:*:*****::****:.:*

H.sapiens DAB2 (NP_001334.2) ILLVDLNSEIDTNQNSLRENPFLTNGITSCSLPRPTPQASFLPENAFSAN 283
X.eiseni  dab2               --------------------------------------------------

H.sapiens DAB2 (NP_001334.2) LNFFPTPNPDPFRDDPFTQPDQSTPSSFDSLKSPDQKKENSSSSSTPLSN 333
X.eiseni  dab2               ---------------------------------------NSGSS------ 248
                                                                    **.**

H.sapiens DAB2 (NP_001334.2) GPLNGDVDYFGQQFDQISNRTGKQEAQAGPWPFSSSQTQPAVRTQNGVSE 383
X.eiseni  dab2               --------------------------------------------------

H.sapiens DAB2 (NP_001334.2) REQNGFSVKSSPNPFVGSPPKGLSIQNGVKQDLESSVQSSPHDSIAIIPP 433
X.eiseni  dab2               --------------------------------------------------

H.sapiens DAB2 (NP_001334.2) PQSTKPGRGRRTAKSSANDLLASDIFAPPVSEPSGQASPTGQPTALQPNP 483
X.eiseni  dab2               ------------------DLFGTDLFVPPVS------SETSPADLFNNTP 274
                                               **:.:*:*.****      * *. .  :: .*

H.sapiens DAB2 (NP_001334.2) LDLFKTSAPAPVGPLVGLGGVTVTLPQAGPWNTASLVFNQSPSMAPGAMM 533
X.eiseni  dab2               TNTVPSTIPALG--SLQLGPTATSVPAVGMWGTSPAVPAMFP--MPGIVT 320
                              : . :: **     : ** .:.::* .* *.*:. *    *   ** :

H.sapiens DAB2 (NP_001334.2) GGQPSGFSQPVIFGT--SPAVSGWNQPSPFAASTPPPVPVVWGPSASVAP 581
X.eiseni  dab2               PGLRPNFPQPTAFGVPMQPPVWAPQVVPQFSAAPLSPPHLQWGQPATS-- 368
                              *  ..*.**. **.  .*.* . :  . *:*:. .*  : ** .*:

H.sapiens DAB2 (NP_001334.2) NAWSTTSPLGNPFQSNIFPAPAVSTQPPSMHSSLLVTPPQPPPRAGPPKD 631
X.eiseni  dab2               ----------NPFQ-------AMGDHGPSRP------PPRPPVKETPPR- 394
                                       ****       *:. : **        **:** :  **:

H.sapiens DAB2 (NP_001334.2) ISSDAFTALDPLGDKEIKDVKEMFKDFQLRQPPAVPARKGEQTSSGTLS- 680
X.eiseni  dab2               VENSAFTALDPLGDKEKKTGKDMFKNFQIAKLPAIPARKGELMPSSTPPP 444
                             :...************ *  *:***:**: : **:******  .*.* .

H.sapiens DAB2 (NP_001334.2) ------AFASYFNSKVGIPQENADHDDFDANQL-LNKINEPPKPAPRQVS 723
X.eiseni  dab2               ANKESVPFDEYFSNKVGLAQDAADHDDFDINQMSILDGNDAPKQTPVQFT 494
                                   .* .**..***:.*: ******* **: : . *:.** :* *.:

H.sapiens DAB2 (NP_001334.2) LPVTKS------TDNAFENP-----------------FFKDSFGSSQASV 750
X.eiseni  dab2               APAAAPSFTPDLLDAAFSSAPVPNSSAPTLGQDLSHDMFDQAFGAPDPNP 544
                              *.: .       * **...                 :*.::**:.:..

H.sapiens DAB2 (NP_001334.2) ASSQPVSS----------EMYRDPFGNPFA 770
X.eiseni  dab2               FGAPPVAMNTVAQTSGSTDAFGDAFGNPFA 574
                              .: **:           : : *.******
```

Figure S4. Amino acid sequence alignment for *Homo sapiens* (*H. sapiens*) DAB2 and *Xenotoca eiseni* (*X. eiseni*) dab2-like proteins.

Journal of Experimental Biology • Supplementary information

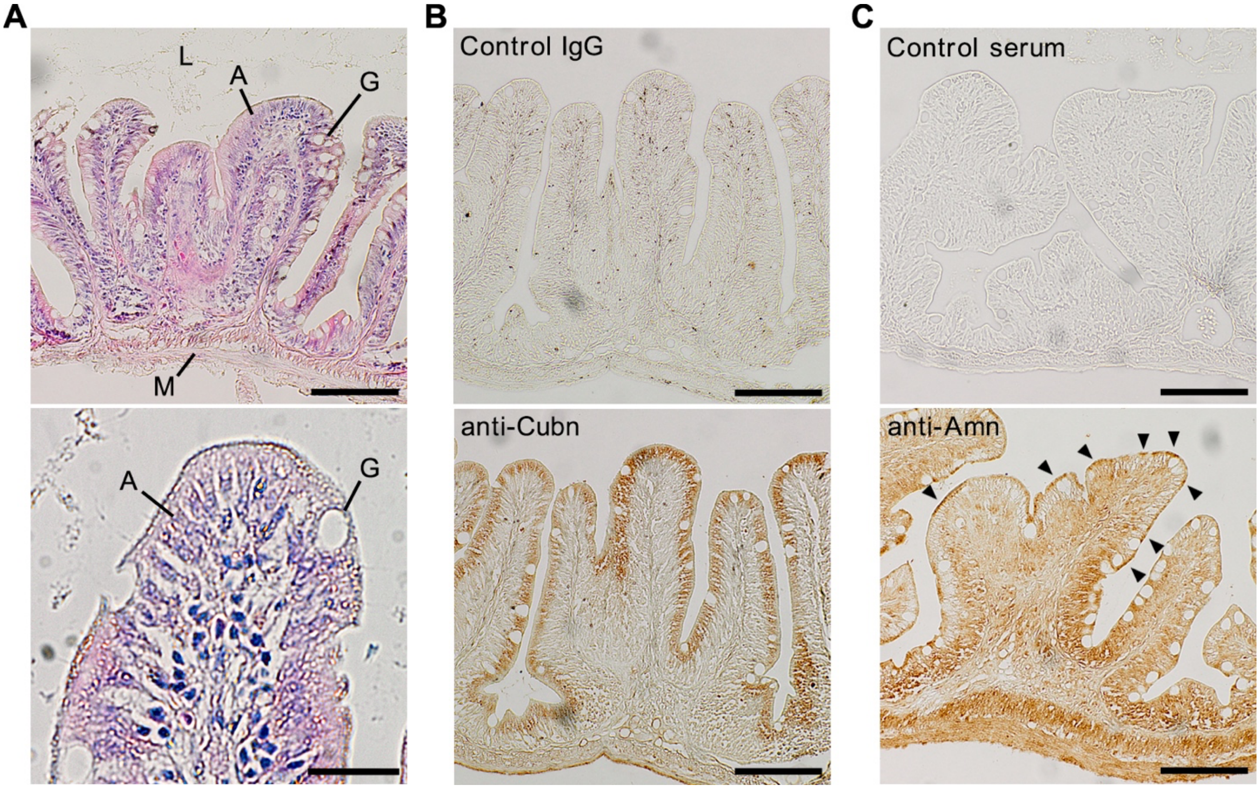

Figure S5. Immunohistochemistry of Cubn or Amn in the adult intestine of *Xenotoca eiseni*.

A. Hematoxylin and eosin-stained section of the hindgut of an adult female. The lower panel is an enlarged image of an intestinal villus. A, absorbent epithelium; G, goblet cell; L, intestinal lumen; M, muscularis mucosae. Scale bar: 100 μm (upper) and 20 μm (lower). **B**. Immunohistochemistry using the Cubn antibody or control IgG. Scale bar: 100 μm. **C**. Immunohistochemistry using the Amn antiserum or control serum. Arrowheads indicate signal in the apical surface of the absorbent epithelium. Scale bar: 100 μm.

Journal of Experimental Biology • Supplementary information

Table S1. List of antibodies used in the study.

Name	Immunogen	Host	Supplier	ID	Related figures
CUBN Polyclonal Antibody (anti-Cubn)	Human CUBN	Rabbit	Invitrogen	PA5-83684	4E, 4G-H, S2A, S3, S5B
Rabbit IgG Isotype Control (Control IgG)	N/A	Rabbit	Invitrogen	02-6102	4E, 4G-H S2A, S3, S5B
Cubn antiserum (anti-Cubn #2)	*X. eiseni* cubn	Mouse	N/A	N/A	S2C-D
Amn antiserum (anti-Amn)	*X. eiseni* amn	Mouse	N/A	N/A	4F, S2B, S5C
Preimmune serum (Control serum)	N/A	Mouse	N/A	N/A	4F, S2B-D, S5C
Anti-Fibronectin antibody	Human FN	Rabbit	Sigma	F3648	4D
Goat anti-Mouse IgG (H+L) Secondary Antibody, Alexa Fluor Plus 488	Rabbit IgG	Goat	Invtrogen	A32723	4G, S3
Horse Anti-Mouse IgG Antibody (H+L), Peroxidase	Rabbit IgG	Horse	VECTOR	PI-2000	4E, S2A, S5B
Goat Anti-Rabbit IgG Antibody (H+L), Peroxidase	Mouse IgG	Goat	VECTOR	PI-1000	4F, S2B-D, S5C
Goat Anti-Rabbit IgG Antibody (H+L), Biotinylated	Rabbit IgG	Goat	VECTOR	BA-1000	4H
Donkey polyclonal anti-rabbit IgG Alexa Fluor® 594 conjugated	Rabbit IgG	Donkey	Thermofisher	A21207	4D

N/A, not applicable.
